# Human salivary Raman fingerprint as biomarker for the diagnosis of Amyotrophic Lateral Sclerosis

**DOI:** 10.1038/s41598-020-67138-8

**Published:** 2020-06-23

**Authors:** C. Carlomagno, P. I. Banfi, A. Gualerzi, S. Picciolini, E. Volpato, M. Meloni, A. Lax, E. Colombo, N. Ticozzi, F. Verde, V. Silani, M. Bedoni

**Affiliations:** 1 IRCCS Fondazione Don Carlo Gnocchi, Milan, 20148 Italy; 2 Department of Psychology, Università Cattolica del Sacro Cuore, Milan, 20123 Italy; 3 Istituto Auxologico Italiano, IRCCS Department of Neurology and Laboratory of Neuroscience, 20149 Milan, Italy; 4 Department of Pathophysiology and Transplantation, “Dino Ferrari” Center, Università degli Studi di Milano, 20122 Milan, Italy; 5 “Aldo Ravelli” Center for Neurotechnology and Experimental Brain Therapeutics, Università degli Studi di Milano, 20122 Milan, Italy

**Keywords:** Diagnostic markers, Neuromuscular disease

## Abstract

Amyotrophic Lateral Sclerosis (ALS) is a neurodegenerative disease leading to progressive and irreversible muscle atrophy. The diagnosis of ALS is time-consuming and complex, with the clinical and neurophysiological evaluation accompanied by monitoring of progression and a long procedure for the discrimination of similar neurodegenerative diseases. The delayed diagnosis strongly slows the potential development of adequate therapies and the time frame for a prompt intervention. The discovery of new biomarkers could improve the disease diagnosis, as well as the therapeutic and rehabilitative effectiveness and monitoring of the pathological progression. In this work saliva collected from 19 patients with ALS, 10 affected by Parkinson’s disease, 10 affected by Alzheimer’s disease and 10 healthy subjects, was analysed using Raman spectroscopy, optimizing the parameters for detailed and reproducible spectra. The statistical multivariate analysis of the data revealed a significant difference between the groups, allowing the discrimination of the disease onset. Correlation of Raman data revealed a direct relationship with paraclinical scores, identifying multifactorial biochemical modifications related to the pathology. The proposed approach showed a promising accuracy in ALS onset discrimination, using a fast and sensitive procedure that can make more efficient the diagnostic procedure and the monitoring of therapeutic and rehabilitative processes in ALS.

## Introduction

Amyotrophic Lateral Sclerosis (ALS) is a complex and lethal neurodegenerative disease that progressively leads to irreversible muscle atrophy due to the death of motoneurons replaced by gliosis, with a life expectation from the onset of first symptoms between 2 and 5 years, depending on the cases^1^. This disorder affects both lower and upper motoneurons with symptoms including generalized muscle weakness, possible cognitive dysfunction, cramps, fasciculations, spasticity, serious functional limitations with parallel and progressive paralysis leading to death, typically resulting from ventilatory failure^2^. An American study showed that there are 223,000 people affected by ALS worldwide with an incidence of 1.75/100,000 and a predicted increase of 69% in 2040 due to the population aging^3^. The causes for ALS disease are still unclear with different mechanisms proposed including genetic, environmental, viral, immunological and epidemiological factors^4^. Compared to other neurodegenerative diseases, the identification of potential biomarkers in ALS has been hampered by the long lag-time between symptoms onset and diagnosis (approximately 12 months) and to the low annual incidence that makes general screening strategies not feasible^5^. Nowadays, no diagnostic test can specifically detect ALS at onset and discriminate ALS from other motoneuron and similar neurodegenerative diseases, thus hindering the diagnosis, prognosis, patients’ stratification, treatment monitoring or the objective evaluation of the effects of new possible therapies. Currently, the diagnosis of ALS is achieved by the combination of clinical data and neurophysiological evidence together with the monitoring of the symptoms progression in a time-consuming process that limits the time frame for a prompt intervention and the choice of a personalized therapy^6^. The discovery of a new biomarker easily accessible and quickly detectable represents a priority for ALS early diagnosis, stratification and evaluation of the therapeutic and rehabilitative effectiveness.

In recent years, several potential biomarkers were isolated from different tissues and highly specific techniques have been proposed. The road taken by researchers regards the analysis of biofluids, whose molecular composition (i.e. proteins, lipids, nucleic acids, carbohydrates, metabolites, hormones) is representative of the physiological/pathological state. The research for biomarkers related to ALS has been performed principally on Cerebrospinal Fluid (CSF), with a large variety of molecules associated with ALS including neurofilament proteins, components of the inflammation process, *C9orf72* dipeptide repeat proteins, TAR DNA-Binding protein (43 kDa), cystatin C, specific microRNA (miRNA181a-5p, miRNA-143-5p, miRNA-338-3p) and the mutated Superoxide Dismutase enzyme type 1 (SOD1)^7^. Some of the listed molecules have been detected also in serum and plasma, that share a less invasive collection procedure compared to CSF, making periodical collection feasible. Overall, no reliable and repeatable data have been obtained, so far^8–11^. Up to now, one of the most promising genetic biomarkers is the mutation of *C9orf72* repeat expansion, which has been attributed to the onset of familial ALS, frontotemporal lobar degeneration and a small part of sporadic ALS^12^. Clinical studies are under development to validate the C9-based therapies, but its restriction to familial ALS (5–10% of the total^13^) and the continuous need for the CSF collection to monitor the disease progression using the *C9orf72* dipeptide repeat proteins are limiting the development of this approach^7^. Other specific biomarkers are under investigation for the detection and discrimination of sporadic ALS (~90% of the cases) from the familial onset, although the two forms possess a comparable pathological mechanism with common biomarkers (e.g. TAR DNA-binding protein)^14^. Similarly, also mutations of SOD1 (~20% of familial ALS) and TAR DNA-binding protein (2–5% of familial ALS) genes, show the same limitations of C9-based proteins regarding the invasiveness of biofluid collection procedures^13,15^. In the same way, neurofilament proteins have been studied as indicator of neurodegeneration in ALS and other neurodegenerative diseases. Different studies reported high levels of neurofilament in CSF and blood of ALS patients and related pathological controls, respect to the healthy counterparts highlighting a neurodegenerative process in progress^8,16–18^.

Despite some promising results, the above cited biomarkers have also fuelled controversies, mainly regarding their specificity for ALS. For example, high levels of neurofilaments and inflammatory mediators are associated to generic axonal injuries and neuroinflammation, that are present both in pathogenic processes occurring in ALS, but also in other neurodegenerative diseases^18,19^. In order to find an univocal correlation with ALS, a pattern of inflammatory molecules (around 248 molecules) is under evaluation^7^, although a fast technique to detect concurrently such a cohort of biomarkers is still missing^20^.

Although CSF and blood-based samples are the most analysed biofluids, the invasiveness of their collection procedure still represents a hardly surmountable obstacle, especially for degeneration and therapy monitoring in late-stage patients with ALS (pALS). For this reason, other biofluids more easily accessible have been investigated including saliva.

Saliva is a complex biofluid composed of different molecules (proteins, metabolites, carbohydrates, nucleic acids and hormones) in an aqueous environment. These molecules undergo active and passive processes of transport from oral cavity cells, salivary glands and plasma to saliva, thus representing potential biomarkers^21^. The concentrations and presence of the salivary molecules are strictly dependent on the pathological state, indicating not only the onset of specific diseases, but also its progression and response to specific pharmacological and rehabilitation treatments. Nowadays, different salivary biomarkers have been proposed and ascertained for the diagnosis of neurodegenerative diseases like Alzheimer’s and Parkinson’s diseases, periodontal pathologies, diabetes, lung cancer, Sjögren’s syndrome, virus and bacterial infections and also for the presence of drugs in the body^22–27^. Regarding ALS, few studies reported the presence of molecules in saliva that can be used as indicators of pathology onset, in particular Chromogranin A (ChA) and cortisol, indicating that deeper studies are needed to completely evaluate the potential of this biofluid^28,29^.

The methodology used was mainly based on proteomic analysis and ELISA assays that rely on expensive procedures and antibody specificity, respectively. Still, the possibility to analyse with a single technique the whole constituents of saliva, instead of a single biomarker, is of crucial importance, in order to identify the differences between healthy subjects and pALS in a rapid way.

Raman Spectroscopy (RS) is a valid technique for the biochemical characterization of biological samples that doesn’t require any label or complex sample preparation. RS is a vibrational spectroscopy, non-destructive, sensitive, rapid and automatable technique providing a spectrum that describes the chemical composition of a sample, potentially avoiding the need for single protein biomarker detection. The output of RS represents an overview of all the molecules contained in a specific biofluid with information regarding presence, concentration, environment, interactions and possible mutations^30^. It has been used for saliva components characterization in the forensic field, but it was also investigated for its potential application in the oncologic field where RS was successfully applied for leukaemia, breast, head, neck and oral cancer with a sensitivity ranging from 75% to 95%^30–33^. One important advantage of RS relies in the existence of highly sensitive portable microRaman spectrometers proposed as biosensing point of care, already studied and assessed for the diagnosis of bacterial infections in CSF and for skin cancer^34,35^. Recently, also a possible role in neurodegenerative diseases diagnosis was proposed for RS due to the method sensitivity^36,37^. In case of specimen in low concentrations or hardly detectable, the RS can be made more sensitive due to the presence of nanostructured metallic surfaces, taking advantage of an effect called Surface Enhanced Raman Scattering (SERS), already used for the analysis of proteins in saliva samples^30^.

In this work, RS has been used for the analysis of saliva collected from 19 pALS and compared with data obtained from saliva collected from 10 Healthy Controls (CTRL), 10 patients affected by Parkinson’s Disease (PD) and 10 affected by Alzheimer Disease and Mild Cognitive Impairment (AD). The acquisition methodology was standardized taking into consideration the effects of laser power, acquisition time, substrates, saliva filtration and SERS inducers. The obtained results demonstrated the possibility to distinguish pALS between the experimental groups through the fast and label-free analysis of saliva, collected with a minimal invasive procedure. The resulting Raman fingerprint represents a “global” biomarker, sensitive to the presence and relative amount of all the molecules contained in salivary samples. Our findings open the way for the development of a new potential diagnostic methodology, potentially able to monitor the therapeutic and rehabilitative effectiveness, with benefits from both the clinical and methodological point of view.

## Results

### Raman analysis optimization

**Figure 1:**
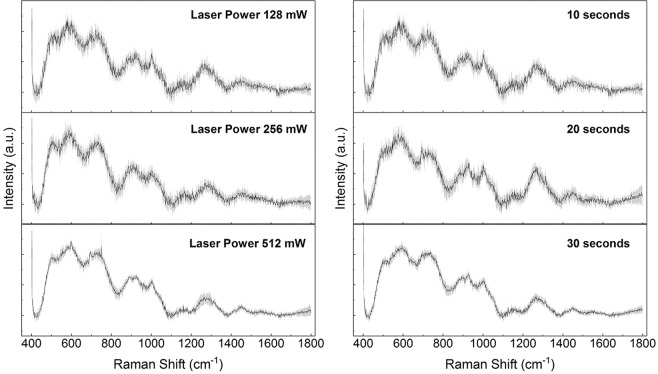
Average Raman spectra (black line) and standard deviation (grey band) of saliva obtained using laser power of 128, 256 and 512 mW (left column) and acquisition time of 10, 20 and 30 seconds (right column).

**Figure 2:**
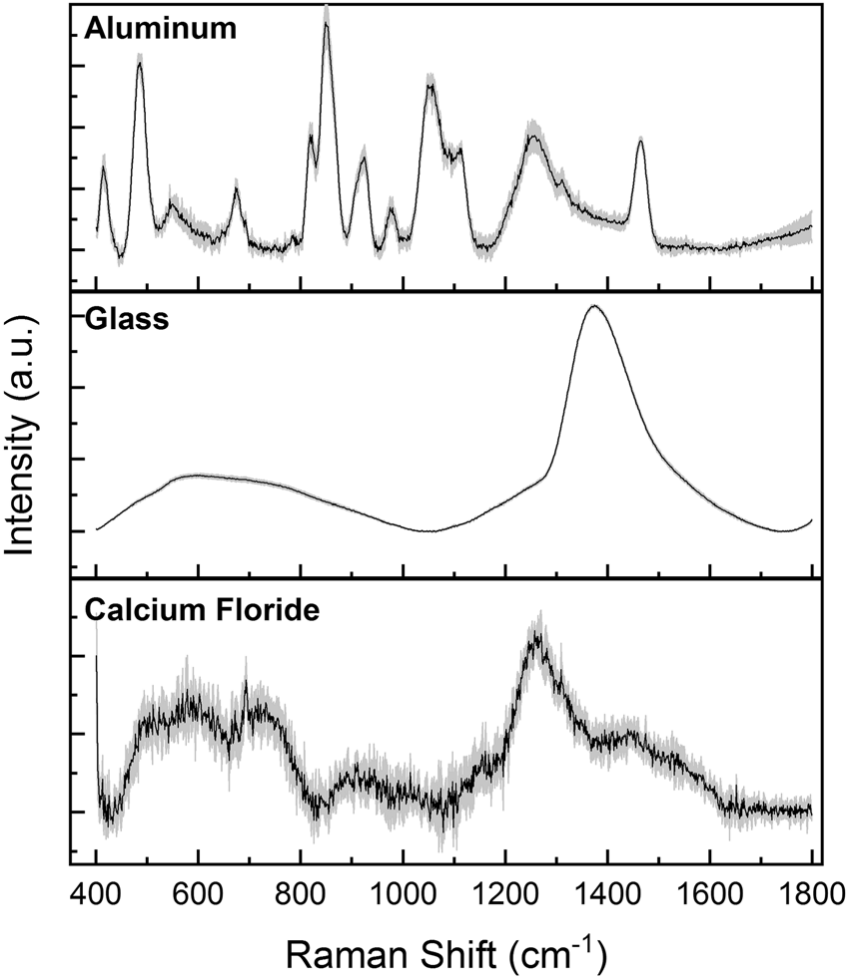
Average Raman spectra of saliva analyzed on aluminium foil, glass and calcium fluoride.

In the first part of the study, we aimed to standardize the methodology for the RS, investigating the effects of analysis parameters and SERS enhancer on the quality of the final saliva spectra, without complex saliva processing. The chosen laser wavelength was 785 nm based on previous experiments reported in literature, being the optimal investigative source for the molecules mixture in saliva^38^. For this reason, we tested different analytical parameters including laser power, acquisition time, Raman substrates, cut-off filters on saliva and the potential application of nanostructured materials and metal surfaces for the SERS induction. Figure 1 shows the effects of laser power (512, 256, 128 mW) and acquisition time (10, 20, 30 seconds) on the final spectra of saliva deposited on CaF_2_ disks. As expected, an increment of both these parameters allowed the collection of more reproducible spectra (lower standard deviation values, SD). Despite the SD reduction, the obtained spectra possessed a low detail level with only few characteristic peaks attributable to specific molecules, including the peak at 1270 cm^−1^ for the phospholipids, 719 cm^−1^ for the nucleic acids and 923 cm^−1^ for the glucose^39^. In order to implement the information obtained from the spectra, we tested different substrates including glass, CaF_2_ and aluminium foils. Compared to glass that partially interfered with the Raman signal, CaF_2_ disks were Raman invisible substrate, while aluminium can be used as SERS inducer due to the metallic composition and the surface roughness. Before the RS analysis, saliva was filtered using standard filters with a cut-off of 3 kDa in order to remove substances, e.g. cell fragments, albumin or protein aggregates, that can influence the Raman spectra and obstacle the formation of the “hot spot” for the SERS signal^40^. In Fig. 2 the spectra obtained using the three substrates are shown. It was clearly visible that the SERS effect induced by the aluminium foil led to a more repeatable and detailed spectrum compared to glass (no signal detected, Fig. 2 Glass) and CaF_2_ (Fig. 2 Calcium Fluoride). Probably, the signal from the glass substrate dominated the entire spectra, being the peak at ~1400 cm^−1^ typical from these typologies of materials^41^. The differences between the aluminium and CaF_2_ were due to the absorption of the salivary molecules on the metallic surface allowing a more detailed spectrum with the only preserved peak at 1261 cm^−1^. The salivary fingerprint showed the well-defined peaks reported in Table 1.

**Table 1:** Identified peaks with an attribution attempt of most relevant molecules based on data reported by Movasaghi *et al*.^75^ and Virkler *et al*.^45^ (±8 cm^−1^).

Raman Shift (cm^−1^)	Attribution
414	Phosphatidylinositol
486	DNA/Glicogen
540–556	Cholesterol, glucose, saccharide and acyl bands
677	Ring breathing modes in DNA bases G
823	Out of plane ring breathing/Tyrosine/Phosphodiester
851	α-Glucose/Polysaccharides
923	C-C stretch of proline ring/Glicogen/Glucose
979	C-C stretching of protein β-sheets
1056	Lipids
1111	Glucose/Lipids/C-C-H bending
1230–1280	Amide III/Phospholipids
1470	Deoxyribose, CH_2_ stretching

Such peaks are comparable with the values reported in literature, with shifts due to the different SERS inducers used in other works where metallic nanoparticles were considered^39,42^.

**Figure 3:**
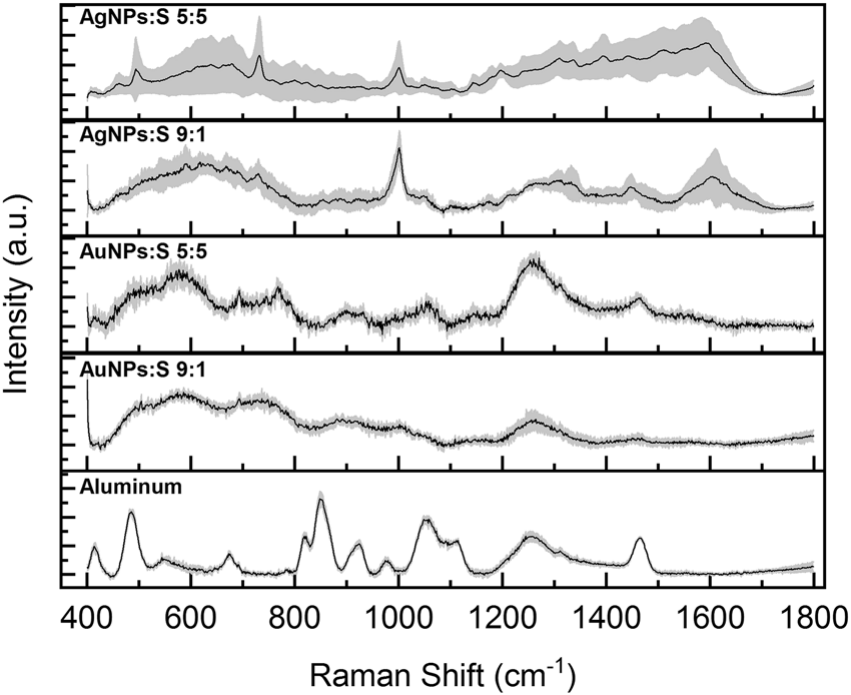
Average Raman spectra of saliva mixed with silver nanoparticles (AgNPs) and gold nanoparticles (AuNPs) with ratios NPs:saliva of 5:5 and 9:1 compared with the one acquired on aluminium foil.

To further evaluate the SERS signal, we analysed the effects of two different metallic nanoparticles in different ratios with filtered saliva, in particular AgNPs and AuNPs with ratios of 5:5 and 9:1, and compared them to the aluminium substrate (Fig. 3). The results for the different concentrations of AgNPs are coherent with other analysis of saliva reported in literature^38^, confirming the possibility to obtain information from the biofluid using AgNPs as SERS inducers. Moreover, an increase in NP concentration led to more detailed spectra, probably due to the formation of protein aggregates and to the complete absorption of proteins on the NP surface. Concerning the use of AuNPs, the final spectra were similar to those obtained from filtered saliva cast on CaF_2_ (Fig. 2), indicating a low or absent SERS signal. Compared to the other nanostructured systems (AgNPs and AuNPs), aluminium foil showed defined peaks and higher reproducibility, probably due to the uniform layer of salivary molecules created after the deposition.

Regarding the application of SERS methods, specific proteins such as albumin, tend to aggregate on NPs surfaces creating a layer of proteins that inhibits the SERS effect. Indeed, these aggregates avoid the NPs aggregation and consequently the formation of the hot spot leading to the loss of SERS signal. A possible approach to overcome this problem is to remove a part of the hindering proteins, for example albumin, leading to the correct interactions between biological molecules and nanostructures^40^. For this reason, the optimal balance between protein content (concentration, type, molecular weight, charge) and NPs has to be experimentally identified for every type of biological sample undergoing SERS analysis in order to obtain a repeatable signal without reducing the sample informative power.

**Figure 4:**
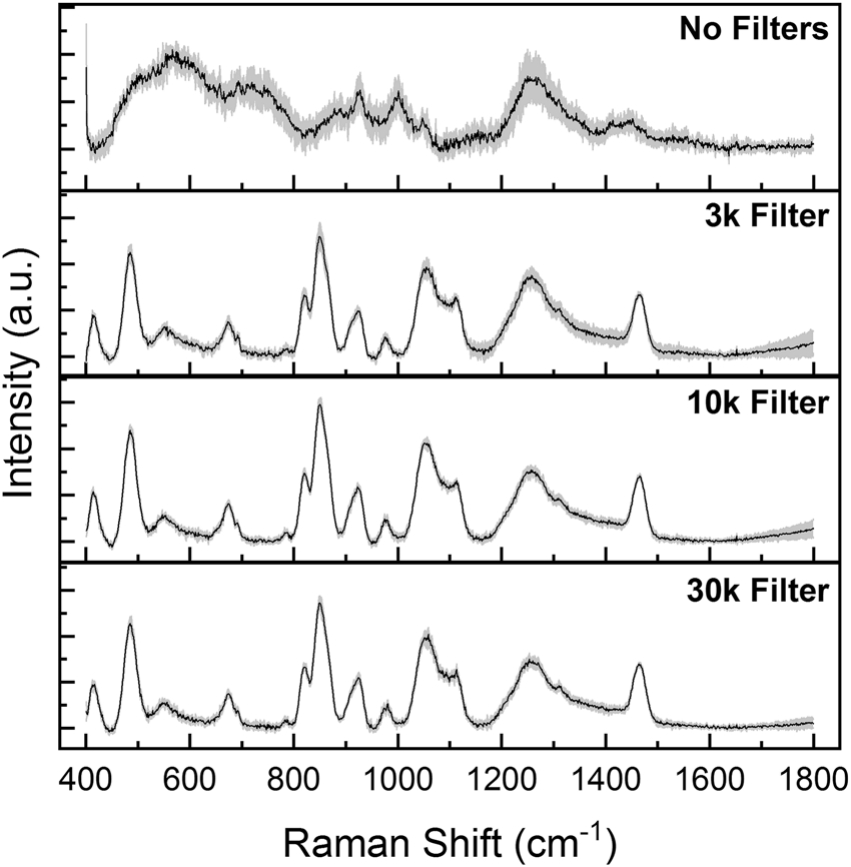
Average Raman spectra of saliva without and after filtering with 3, 10, 30 kDa cut-offs.

Herein, we tested different filters with increasing values of cut-off (3, 10 and 30 kDa) evaluating the possible loss of information due to the protein retention. In Fig. 4 the four spectra related to pure saliva and to saliva filtered with 3, 10 and 30 kDa filters, deposited on the aluminium foil are reported, demonstrating that the filtration process did not substantially affect the final information, resulting in similar spectra without remarkable differences. Therefore, for the further analyses, we decided to use 3 kDa filters able to remove those molecules that avoid the SERS effect and, at the same time, preserve information given by the small molecules in saliva. As result from the methodological part, we obtained the optimal parameters for the analysis of saliva using an aluminium foil as substrate after biofluid filtration with a 3 kDa cut-off.

### Raman analysis of clinical samples

**Figure 5:**
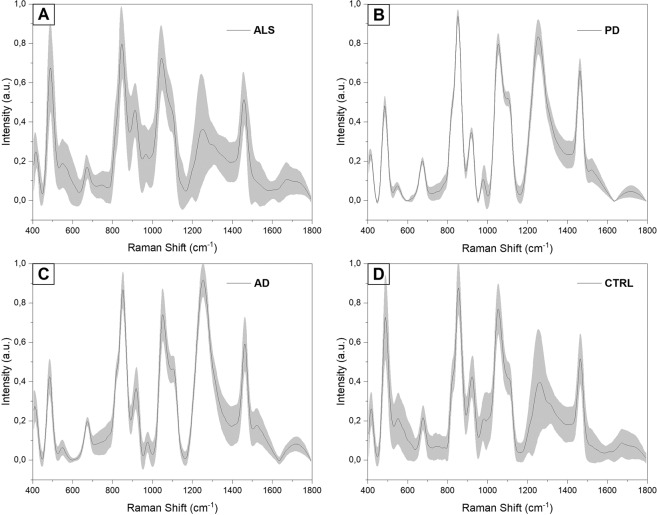
Average Raman spectra with SD of (**A**) ALS, (**B**) PD, (**C**) AD and (**D**) CTRL groups.

**Figure 6:**
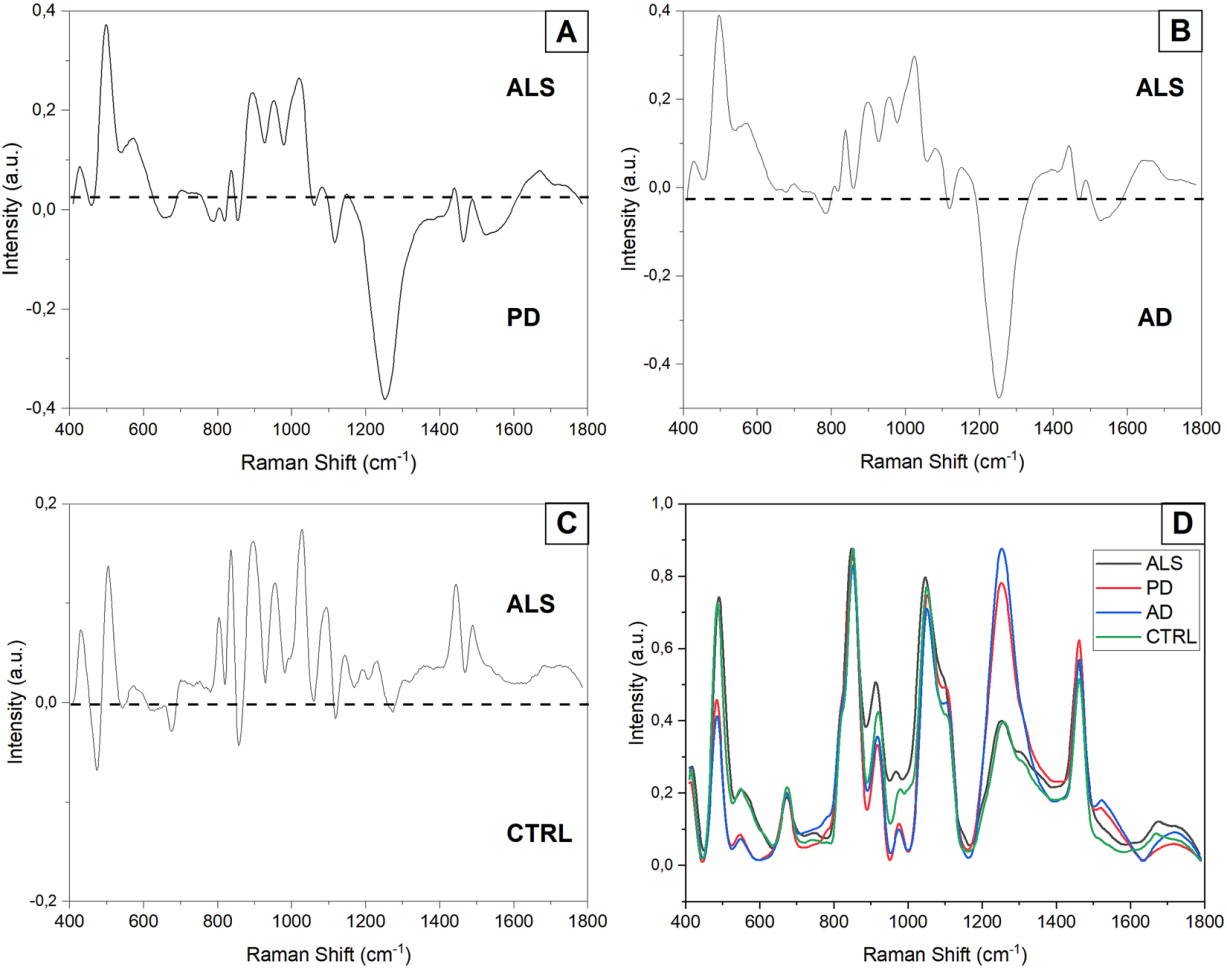
Subtraction spectra of the average ALS signal versus the (**A**) PD average signal, (**B**) AD average signal and (**C**) CTRL average signal. (**D**) Overlapped average spectra of the experimental groups.

In the present study we used the previously optimized parameters to analyse the saliva collected from 19 pALS, 10 PD, 10 AD and 10 CTRL. In Fig. 5, the average SERS spectra obtained from all the experimental groups are shown (Fig. 5A) ALS, B) PD, C) AD and D) CTRL). As it is possible to notice, the main differences were due to intensity variations of defined peaks suggesting changes in the concentration of specific biomolecules present in the saliva of the examined groups. The principal differences of the ALS group respect to the two pathological controls (Fig. 6A,B) PD and AD can be find in peaks at 430, 500, 576, 833, 890, 951, 1021, 1120, 1251, 1470, 1540 and 1670 cm^−1^ (Δ Intensity ≥0.1). Analysing the differences between the calculated spectral means of ALS and CTRL (Fig. 6C), the main intensity changes were due to the bands at 500, 833, 890, 923, 1021, 1445 cm^−1^ (Δ Intensity >0.1). Other spectral differences were identified at 430, 472, 576, 677, 808, 1120, 1192, 1231, 1470 cm^−1^ (Δ Intensity <0.1). The intensity differences identified can be clearly visible in the overlapped average spectra collected from each experimental group, where also no or slight peak shifts were detected (Fig. 6D, ∆shift ≤3 cm^−1^).

**Figure 7:**
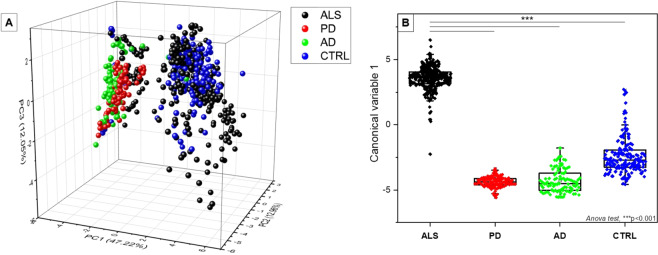
(**A**) Principal Component Analysis (PCA) 3 axis distribution (X = PC1; Z = PC2; Y = PC3). (**B**) Linear discriminant Analysis (LDA) showing the distribution of canonical variable values for the ALS (n = 19), PD (n = 10), AD (n = 10) and CTRL (n = 10). ***p < 0.001, One-Way ANOVA test.

To verify the observed differences, we used a MultiVariate statistical Analysis (MVA) to build a classification model for the discrimination between ALS, AD, PD and CTRL. The PCA- LDA analysis was performed on all the collected data (Fig. 7). The score plot in Fig. 7A allows to easily visualize how the first three Principal Components (PCs) obtained from PCA, with the highest loads (PC1 = 47.22%, PC2 = 12.96% and PC3 = 12.05%), describe the main spectral differences between the four groups. As highlighted by the graph of data dispersion, the CTRL and ALS regions were partially overlapped with intra-regions focus points where PCs values are concentrated. Similarly, also the dispersions of PCs for the pathological controls, AD and PD, were partially overlapped, with the focus points well separated from the ALS and CTRL one. The first 10 PC scores were then used to perform the LDA analysis as summarized in Fig. 7B. The dispersion of the ALS Canonical Variable (CV) values was proved to be statistically different from the other three groups (p < 0.001, One-Way ANOVA test) indicating that RS analysis is able to distinguish the spectra acquired from the pALS saliva. The error rate after the cross-validation of training data was 7.37%, while, after confusion matrix analysis, the method showed accuracy, precision, sensibility and sensitivity to detect saliva of pALS between all the data of more than 98% for all the parameters.

### Correlation

Data obtained from the MVA, in particular CV, PC1, PC2 and PC3, were correlated with clinical and behavioral aspects of pALS, ascertainable during the saliva collection procedure, including patients age, smoking habit, dysphagia degree, PEG, time from the diagnosis and from the last meal, IMV, ALS-FRS, WHO-QOL scores, ECAS scores, blood pH, oxygen and carbon dioxide partial pressure (Table 2).

**Table 2:** Clinical and behavioral parameters with standard deviation of pALS collected for the Raman data correlation. In brackets the number of patients considered for the corresponding parameter.

Collected Data	Mean ± Standard Deviation
Age (n = 19)	74.6 ± 5.6
Smoking Habits (n = 14)	yes/not/ex
Dysphagia (DOSS Degree) (n = 14)	3.7 ± 2
Percutaneous Endoscopic Gastrostomy (n = 14)	yes/not
Time from the Diagnosis (months) (n = 19)	69.7 ± 62.7
Invasive Mechanical Ventilation (n = 14)	yes/not
Time from the Last Meal (minutes) (n = 14)	145 ± 47.1
ALS - Functional Rating Scale (n = 19)	16 ± 6.7
World Health Organization - Quality of Life (n = 14)	82 ± 9.2
Edinburgh Cognitive and Behavioural ALS Screen (n = 19)	74.6 ± 23.7
Blood pH (n = 14)	7.43 ± 0.03
Oxygen partial pressure (n = 14)	76.2 ± 17.3
Carbon Dioxide partial pressure (n = 14)	40.6 ± 4.4

The independence of the method from environmental and clinical factors that can potentially influence the saliva analysis was confirmed by the missing correlation of CV, PC1, PC2 and PC3 with patients age, smoking habits, IMV and the time spent from the last meal before the saliva collection, where not statistically significant Pearson’s coefficients were always obtained (Table 3). The missing correlation of these parameters with our data indicated the reliability of the Raman analysis of saliva, being not influenced by parameters which can potentially alter the biochemical composition of the biofluid.

**Table 3:** Pearson’s coefficients and relative p-values of Canonical Variable (CV), Principal Component 1 (PC1), 2 (PC2) and 3 (PC3) correlated with Edinburgh Cognitive and Behavioral ALS Screen (ECAS) score, patients age (Age), ALS- Functional Rating Scale (ALS-FRS), World Health Organization - Quality Of Life (WHO-QOL), time from the diagnosis expressed in months (Diagnosis), time from the last meal expressed in minutes (Meal), Invasive Mechanical Ventilation (IMV), blood pH (pH), oxygen (PaO_2_) and carbon dioxide partial pressure (PaCO_2_), smoking habit (smoke), Percutaneous Endoscopic Gastrostomy (PEG) and dysphagia degree (DOSS).

		ECAS	Age	ALS-FRS	WHO-QOL	Diagnosis	Meal	IMV	pH	PaO_2_	PaCO_2_	Smoke	PEG	DOSS
CV1	**Pearson**	0,37047	0,21649	0,05751	0,47013	−0,35528	−0,01699	0,46416	0,25035	−0,11791	−0,34783	0,12469	0,15427	0,0675
	**p-value**	0,11844	0,40395	0,83246	0,077	0,14795	0,9502	0,15038	0,45779	0,7299	0,29456	0,69942	0,61482	0,8349
PC1	**Pearson**	−0,04577	−0,17697	0,77874*	0,21052	−0,0349	0,40255	−0,33238	−0,02795	0,15328	−0,16333	0,22035	0,02531	0,10794
	**p-value**	0,85241	0,49684	**3,79E-04**	0,45139	0,89066	0,12214	0,31795	0,935	0,65274	0,63134	0,49134	0,93458	0,73845
PC2	**Pearson**	0,16409	0,30449	0,19251	0,86327*	−0,08801	−0,22129	0,21309	0,44248	−0,12762	−0,51816	0,06822	0,13213	0,0528
	**p-value**	0,50205	0,23472	0,47504	**3,40E-05**	0,7284	0,41014	0,52929	0,17294	0,70846	0,10251	0,83314	0,66698	0,87054
PC3	**Pearson**	0,68096*	0,22883	−0,00528	−0,16432	−0,53948*	0,1509	0,53681	0,39122	−0,32469	−0,33661	−0,47564	−0,17633	−0,26191
	**p-value**	**0,00133**	0,37701	0,98451	0,55841	**0,02085**	0,57695	0,08864	0,23414	0,32996	0,31145	0,11808	0,56445	0,41089

*p < 0.05, Pearson’s test.

Interestingly, the statistically significant coefficients (p < 0.05) were obtained for PC1 and PC2, with ALS-FRS and WHO-QOL scores, and for PC3 with ECAS and time from the diagnosis, with values of Pearson’s coefficient of 0.778 (PC1 - ALS-FRS), 0.863 (PC2 - WHO-QOL), 0.68 (PC3 - ECAS) and 0.539 (PC3 - time from the diagnosis) respectively (Table 3). The PCs represent independent directions, with their own specific weights (loadings), used to maximize the variance between the variables examined during the PCA, in this case PC1 with a loading of the 47.22%, PC2 with the 12.96% and PC3 with 12.05% (Fig. 7A)^43^.

## Discussion

In this work we have optimized the RS parameters, including laser power, acquisition time, substrates and filters with different cut-off, for the analysis of human saliva samples. Afterwards, we have investigated the potentiality of different SERS inducers to improve the reproducibility and intensity of saliva spectra, identifying aluminium as the ideal substrate. The assessed procedure was used for the analysis of saliva from pALS, patients with AD, PD and healthy subjects, evidencing intensity differences between the considered groups. We report, for the first time, the RS analysis of saliva used as potential diagnostic tool for the discrimination of ALS onset. Previously, different studies proposed RS as powerful tool for the fast and sensitive characterization of neurological diseases, being associated to the production of a “whole biomarker” containing information about the complete biochemical composition of the chosen biofluid^44^. These features could provide an effective alternative to the major and enduring healthcare problems for neurodegenerative diseases including the time-consuming diagnostic processes and the costs for the patients. One important example is given by ALS in which the complex diagnostic process can take up to 18 months drastically reducing the time for a prompt therapeutic intervention^5^. The discovery of an easily collectable biomarker could represent a solution for diagnostic/screening/predictive/monitoring purposes, allowing at the same time a deeper understanding of the pathological mechanism. In our work, the implementation of the spectral detail, leaded by the introduction of aluminium foils used as Raman substrate and by an optimized analysis protocol, allowed the discrimination of different peaks attributed to molecules involved in pathological mechanisms. Regarding the differences highlighted between the ALS average signal and the two pathological controls PD and AD (Fig. 6 A,B), the principal peaks can be attributed to differences in concentration of lipids, particularly regarding phospholipids (833, 1251 and 1470 cm^−1^), cholesterol (430 cm^−1^) and phosphatidylinositol (500 and 576 cm^−1^), while all the other differences can be attributed to different protein vibrational modes^45^. The main differences in this confrontation can be attributed to structural and signaling lipids with a strong confirmation in different studies, where is highlighted an altered response in the free radical oxygen species protection^46–49^. Observing the data reported in Fig. 6A,B also an increased presence of cholesterol can be encountered in ALS group respect to AD and PD. A possible interpretation could be find in cholesterol accumulation observed in ALS^50^ and lower concentration of low-density lipoprotein cholesterol noticed in PD^51^. Regarding AD, a correlation between high concentration of cholesterol and the pathology onset has been proposed in literature^52^. The reason of the lower level of cholesterol in AD respect to the ALS signal, could be find in the dietary prescription assigned to the recruited AD patients, which includes different statins for the cholesterol level control.

A further confirmation of the obtained data with the metabolic disorders encountered in ALS, can be find in the attribution of peaks related to the phosphatidylinositol vibrational modes (500 and 576 cm^−1^), which demonstrate higher levels of these lipid molecules in the ALS group (Fig. 6A,B) due to the increased activity of Phosphatidylinositol 3-kinase enzyme in pALS^53^. Regarding the differences encountered in the subtraction spectrum of ALS and CTRL group (Fig. 6C), the principal differences are related to nucleic acids (472, 677, 808 cm^−1^), glycogen and glucose (923 and 1021 cm^−1^) and to lipids, specifically to phospholipids (1231, 1445 and 1470 cm^−1^) and phosphatidylinositol (500 and 576 cm^−1^). These results were coherent with previously reported data, where a change in these molecule levels has been detected in pALS and related to an alteration in carbohydrates metabolism (for glucose and glycogen) and to damages on proteins, nucleic acids and membrane phospholipids possibly caused by free radical oxygen species accumulated as result of mutations in SOD-1^54–56^ and to the dysregulation of Phosphatidylinositol 3-kinase mentioned above. The spectral differences were also evaluated using MVA in order to extract more information from the collected data (Fig. 7). Interestingly, the distribution of the PCs and CV values obtained from the four groups, presented a statistical difference respect to the pALS group, allowing the precise discrimination of the signal coming from the saliva collected from the pALS. The concomitant correlation of the data obtained from the MVA with behavioural, paraclinical and clinical values gave more information about the RS on saliva. First of all, the missing correlation of PC1, PC2, PC3 and CV with age, smoking habits, IMV, PEG and time from the last meal, indicates that RS is not influenced by factors which can potentially modify the biochemical composition of saliva. The PC1 values correlated with ALS-FRS scores (Table 3) obtained from pALS, which take into consideration a series of clinical and behavioral parameters evaluated by the patient itself, including difficulties in respiration, nutrition, physical health, level of independence and movements. All these parameters, as well as the final scores, represent an overview of the subjective clinical state of the patient^57^. As described previously, RS is able to detect the overall biochemical composition of saliva and the positive correlation with ALS-FRS could regard a series of neurological, metabolic and musculoskeletal factors, that are reflected in the biofluid biochemical modifications^58^. A refinement of the Raman methodology on a larger cohort of patients could lead to the discrimination of single or multiple clinical factors which could be able to directly influence the PC and thus individuating in this way the factor that influences most the saliva biochemical composition. Moreover, a comparison with other neurodegenerative diseases, as well as, the definition of the genetic mutations associated with the analyzed pALS could make more specific the association between the biomarker and the ALS pathology. These kind of implementations and results, could lead to the determination of a easily measurable biological characteristic that is directly associated to normal or pathological processes or to a response to therapeutic or rehabilitative interventions, following the recent guidelines proposed by van den Berg *et al*.^59^ about the design and implementation of ALS clinical studies. Regarding the correlation of PC2 (Table 3), the WHO-QOL represents a measurement of the quality of life related to health care, evaluating person’s physical health, psychological state and personal belief. In case of diseases onset, the quality of life suffers of a drastic decrease, especially for disabling events such as ALS. A possible explanation for the PC2-WHO-QOL correlation could be found in the release into saliva of proteins related to the mental stress. Indeed, it has been demonstrated that different proteins can be assumed as “salivary stress biomarkers”, e.g. cortisol, chromogranin A and immunoglobulin A^60^. In particular, cortisol and ChA have been already proposed as potential ALS biomarkers, highlighting the role of these molecules in ALS onset and progression, strictly correlated with stress levels induced by the pathology^28,29,60^. In fact, ALS leads to an unrelenting decrease in patients’ quality of life accompanied to an increase in mental stress levels that might explain the correlation between PC2 and WHO-QOL. The third PC showed a direct correlation with two parameters strictly associated: the ECAS score and the time from the diagnosis (Table 3). The ECAS questionnaire is aimed to determine the multi-domain neuropsychological screening that assesses executive function, social cognition, verbal fluency and language (ALS-specific), as well as memory and visuospatial abilities in pALS^61^ with the final score strictly associated with the pathological progression^62^. The PC3 correlation with these two parameters can be potentially associated to an index able to determine the overall cognitive and executive degeneration evaluating at the same time the pathological progression. In the same way, an enhancement of this correlation on a larger cohort of patients could be a powerful monitoring tool to assess the respiratory and cognitive rehabilitation in pALS.

In order to deepen the molecular bases of these relationships and validate the correlation between PCs with these paraclinical tests, further studies on a larger cohort of pALS are needed. In conclusion, our data demonstrated the sensitivity of this SERS based technique and its ability to identify and analyse different molecules at the same time in a biological fluid. The reported MVA was able to detect statistical differences (p < 0.001) between the spectra of pALS respect to CTRL, PD and AD, assessing the potential of RS to be used as fast diagnostic tool for ALS disease and proposing the biochemical fingerprint of saliva as a complex biomarker, obtained with a minimally invasive procedure. Previous studies have already demonstrated the potential of RS as diagnostic, prognostic and therapeutics/rehabilitative monitoring tool for different neurodegenerative diseases proposing an alternative fast and sensitive process for the diagnosis^44^. Related to ALS, the diagnostic process is still complex and time-consuming, limiting precocious intervention and development of new potential therapies. The acquisition of a single spectrum takes between 10 and 30 seconds, depending on the analysis parameters, explaining the potential of the method if compared with the current diagnostic method. This process efficiency on a minimal invasive collected sample could be exploited also for the monitoring of pathological state through the analysis of biochemical modifications for different ALS progressive states and forms, as well as, for the monitoring of therapies and rehabilitation efficacy. The proposed label free strategy based on the SERS analysis of saliva could provide clinicians and researchers with a powerful tool for ALS early diagnosis, personalization, and fine tuning of the different therapeutic and rehabilitative approaches.

## Materials and Methods

### Materials

All the materials and chemicals were purchased from Sigma Aldrich (USA), if not differently specified, and used without further purification steps. Gold Nanoparticles (AuNPs) were synthesized following the Frens’s method^63^. Briefly, a 200 mL water solution of 0.01 wt% tetrachloroauric acid trihydrate (HAuCl_4_ 3H_2_O) was heated until boiling under stirring. Then, 1.4 mL of sodium citrate was quickly added and the solution was cooled at room temperature. Silver Nanoparticles (AgNPs) were synthesized using Lee-Meisel’s protocol^64^. In brief, 45 mg of silver nitrate (AgNO_3_) were dissolved in 250 mL of deionized water and heated until boiling. Then, 5 mL of sodium citrate 1 wt% were added dropwise with the solution under vigorous stirring for 1 hour. Both the nanoparticle solutions were stored at 4 °C. The Raman substrate of Calcium Fluoride (CaF_2_) were purchased from Crystran (UK), while Salivette^®^ for the collection of saliva samples were purchased from Sarstedt (Germany). Commercially available aluminium foils were used as received. Filter with different cut-off ranges (3 kDa, 10 kDa and 30 kDa, Amicon Ultra) were purchased from Sigma-Aldrich (USA). All the materials were used following the manufacturer’s instructions.

### Patients selection

#### Inclusion and exclusion criteria

pALS were recruited if they had previously received a diagnosis according to the El Escorial criteria^65^ and they were male between 50 and 85 years old at entry.

Exclusion criteria for pALS were represented by concomitant obstructive respiratory diseases; renal failure; cardiovascular, oncological, immune, hematological and psychiatric diseases; bacterial or fungal infections in progress (e.g. oral candidiasis); female sex. CTRL were considered if they did not constantly and continuously take drugs (e.g. anti-hypertensive and anti-diabetic drugs) and did not report chronic and inflammatory diseases, particularly the oral cavity. Furthermore, the pathological and not pathological control groups were strictly age matched to pALS and only male subjects were recruited to limit sex hormone variability in saliva. It is well known that the chemical composition of saliva is influenced by the presence of hormones and how this affects a different Raman signature of the saliva of both men and women^66^. 5 pALS were under riluzone treatment during the sample collection. The inclusion criteria for the PD patients were a diagnosis of PD according to the Movement Disorder Society Clinical Diagnostic Criteria for PD^67^. Exclusion criteria included vascular parkinsonism (defined by evidence of relevant cerebrovascular disease, as indicated by brain imaging computed tomography (CT) or magnetic resonance imaging (MRI), or by the presence of focal signs or symptoms that are consistent with stroke); brain tumor; drug-induced parkinsonism (neuroleptic treatment at onset of symptoms); other known or suspected causes of parkinsonism (e.g. metabolic, etc.), or any suggestive features of a diagnosis of atypical parkinsonism; severe speech problems and poor general health; concomitant neurologic and/or psychiatric diseases. For PD patients the clinical evaluation included the quantification of the disease stage with H&Y and the assessment of the symptom severity with MDS-UPDRS motor part III performed by an experienced neurologist. For the AD experimental group, inclusion criteria were the diagnosis of dementia due to Alzheimer’s disease following the McKhann *et al*. guidelines^68^ and diagnosis of Mild Cognitive Impairment (MCI) due to Alzheimer’s Disease following the clinical criteria described by Albert *et al*.^69^. Exclusion criteria were the presence of neurological or major psychiatric comorbidities.

#### Selection process

pALS, CTRL and PD study participants, who consecutively accessed to the IRCCS Fondazione Don Carlo Gnocchi, in Milan (Italy) between 18^th^ October 2017 and 22^nd^ December 2020, were recruited. AD patients were recruited at Istituto Auxologico Italiano, IRCCS Department of Neurology and Laboratory of Neuroscience. 19 ALS patients (n = 19), 10 CTRL (n = 10), 10 PD patients (n = 10) and 10 AD patients (n = 10) met the relevant eligibility criteria for being included. All participants provided written informed consent and the study was approved by the institutional review board at IRCCS Fondazione Don Carlo Gnocchi on 12^th^ March 2018.

#### Instruments

Demographic information as well as clinical data (age and comorbidities) were collected for all the participants. Other personal information, including smoking habits, were collected when ascertainable (Table 2, numbers in brackets). Moreover, the following data were gathered for the pALS: time from the diagnosis, usage of Non Invasive Ventilation (NIV), presence of tracheostomy or Invasive Mechanical Ventilation (IMV), dysphagia (Dysphagia Outcome Severity Scale, DOSS)^70,71^ and the presence of Percutaneous Endoscopic Gastrostomy (PEG).

Disease status was assessed with ALS Functional Rating Scale-Revised (ALS-FRS)^57^, which is characterised by 12 items (0–48 scores). The factors of ALS-FRS correspond to fine motor (coordinated, mostly upper, limb motions (e.g. writing, feeding, dressing and turning)); bulbar function (e.g. speech, swallowing, salivation); gross motor (less finely controlled activities (e.g. turning, dressing, walking, climbing)) and respiratory function (e.g. dyspnoea, orthopnoea, respiratory insufficiency). Respiratory functioning was assessed with spirometry and arterial blood gases (ABG).

ALS participants were assessed thanks to the Edinburgh and Cognitive Assessment Screening (ECAS)^61^, which is an ALS-designed measure of cognitive and behavioural functioning. Executive, language, and verbal fluency domains are described as ALS Specific functions, while the memory and visuospatial domains are described as ALS Non-Specific. The ALS-Specific and ALS Non-Specific domains combine to generate a measure of global cognitive functioning, namely, the ECAS Total score. Wherever possible, participants were encouraged to respond using spoken responses to minimize testing time. Patients with marked dysarthria were allowed to write responses.

Finally, quality of life was assessed in a part of the participants, through the administration of the WHO-QOL, an abbreviated 26-item version of the WHOQOL-100^72,73^. This questionnaire allows to assess four domains: physical (physical health and level of independence), psychological (including spirituality, religion, and personal beliefs), social relationships, and environment. WHO-QOL instruments can be used in a variety of settings, and results are comparable across cultures.

### Sample collection

Saliva collection was performed following the manufacturer’s instructions. To limit variability in salivary content not related to ALS, saliva was obtained from all subjects at a fixed time, after an appropriate lag time from feeding and teeth brushing. Pre-analytical parameters (i.e. storage temperature and time between collection and processing), dietary and smoking habit (when provided) were properly recorded. Briefly, the swab was removed, placed in the mouth and chewed for 60 seconds to stimulate salivation. Then the swab was centrifuged for 2 minutes at 1,000 g. Collected samples were stored at −80 °C. Before the Raman acquisition, saliva samples were filtered with different cut-off ranges, collecting and analysing by RS the eluted sample and discarding the concentrated counterpart.

### Raman and SERS measurements

Raman and SERS spectra were acquired using an Aramis Raman microscope (Horiba Jobin-Yvon, France) equipped with a laser light source operating at 785 nm with laser power ranging from 25–100%. Acquisition time between 10-30 seconds were evaluated. The instrument was calibrated before each analysis using the reference band of silicon at 520.7 cm^−1^. A drop of saliva (3 µL) was dropped on the designed substrate (Glass, Calcium Fluoride or Aluminium foil) and dried at room temperature. Raman spectra were collected from at least 10 points following a line-map from the edge to the centre of the drop. Spectra were acquired in the region between 400 and 1800 cm^−1^ using a 50x objective (Olympus, Japan). Spectra resolution is about 1.2 cm^−1^. The software package LabSpec 6 (Horiba Jobin-Yvon, France) was used for map design and the acquisition of spectra. SERS measurements were performed mixing saliva samples with AuNPs and AgNPs with a variable ratio (1:9 and 5:5) and incubated for 30 minutes before casting. The effect of the different substrates, including aluminium foil to induce the SERS effect, was evaluated by simply casting saliva on the substrate, as explained by Muro *et al*.^66^. All the methods described in this work were performed in accordance with the relevant guidelines and regulations.

### Data processing and statistical analysis

All the acquired spectra were fit with a fifth-degree polynomial baseline and normalized by unit vector using the dedicated software LabSpec 6. The contribution of the substrate was removed from each spectrum. The statistical analysis to validate the method, was performed as described by Gualerzi *et al*.^74^. Principal Component analysis (PCA) was performed in order to reduce data dimensions and to evidence major trends. The first 20 resultant Principal Components (PCs) were used in a classification model, Linear Discriminant Analysis (LDA), to discriminate the data maximizing the variance between CTRL and pALS groups. The smallest number of PCs was selected to prevent data overfitting. Leave-one-out cross-validation and confusion matrix test were used to evaluate the method sensitivity, precision and accuracy of the LDA model. Mann-Whitney was performed on PCs scores to verify the differences statistically relevant between the analysed groups. Correlation and partial correlation analysis were performed using the Pearson’s test, assuming as valid correlation only the coefficients with a p-value lower than 0.05. The statistical analysis was performed using Origin2018 (OriginLab, USA).

### Ethics approval and consent to participate

The collection procedures and data managing were approved in 12/03/2018 by the Ethics Committee of Fondazione Don Carlo Gnocchi with protocol number: 9/2018/CE_FdG/SA.

## Data Availability

All the data and results are included in the manuscript.
